# Surface modification of pH-responsive poly(2-(tert-butylamino)ethyl methacrylate) brushes grafted on mesoporous silica nanoparticles

**DOI:** 10.1080/15685551.2019.1699727

**Published:** 2019-12-11

**Authors:** Abdullah M. Alswieleh, Mufleh M. Alshahrani, Khalid E. Alzahrani, Hamdan S. Alghamdi, Abdurahman A Niazy, Abdulilah S Alsilme, Abeer M. Beagan, Bayan M. Alsheheri, Abdulaziz A. Alghamdi, Mohammed S. Almeataq

**Affiliations:** a Department of Chemistry, College of Science, King Saud University, Riyadh, Kingdom of Saudi Arabia; b Department of Physics and Astronomy, College of Science, King Saud University, Riyadh, Kingdom of Saudi Arabia; c King Abdullah Institute for Nanotechnology, King Saud University, Riyadh, Kingdom of Saudi Arabia; d Prince Naif Health Research Center, Molecular and Cell Biology Laboratory, College of Dentistry, King Saud University, Riyadh, Kingdom of Saudi Arabia; e Department of Oral Medicine and Diagnostic Sciences, College of Dentistry, King Saud University, Riyadh, Kingdom of Saudi Arabia; f King Abdulaziz City for Science and Technology, Riyadh, Kingdom of Saudi Arabia

**Keywords:** Mesoporous silica nanoparticles, polymer brushes, pH responsive polymer, surface-initiated atom transfer radical polymerization

## Abstract

Poly(2‑(tert-butylamino)ethyl methacrylate) brushes (PTBAEMA) are grown from mesoporous silica nanoparticles via surface-initiated atom transfer radical polymerization (SI-ATRP). Linear PTBAEMA brushes are protonated and highly swollen at low pH; brushes are collapsed at pH higher than 7.7 due to deprotonation, as determined by dynamic light scattering (DLS). Quaternization of these brushes is conducted using 2-iodoethanol in alkali media. DLS measurement of nanoparticles shows that surface-confined quaternization occurs and produces pH-responsive brushes with a hydrophobic upper surface. Variation of the 2-iodoethanol reaction time enables the mean degree of surface quaternization. The pH-responsive behaviour of quaternized PTBEAMA brushes at 1 h reaction time indicates low degrees of surface quaternization, dictated by the spatial location of 2-iodoethanol. Almost uniformly quaternized brushes prepared when the conducted for 3 h and became less swollen at low pH than brushes that conducted for 1 h. The intensity of the C − C − O component (286.5 eV) in the C1s X-ray photoelectron spectrum increased, suggesting that the reaction with iodoethanol was successful occurred.

## Introduction

There has been an increase in research on mesoporous silica nanoparticles (MSNs) during the last decades [1–5]. MSNs have been used as promising materials for drug/gene delivery and many other important applications, due to their unique features such as high surface area, large pore volume, excellent physicochemical stability, and facile modification [4–8]. One strategy was to modify the surface of MSNs with polymers [9–13].

A polymer brush consists of one end tethered to a surface[14]. Brushes can be grafted from either planar [15,16] or colloidal [17,18] surfaces using living radical polymerization techniques [19,20]. Depending on the chemical composition, the conformation of polymer brushes can be changed using external stimuli such as temperature [21–23], and solvents [23–25], and pH [23,26–28]. For example, Liu et al. reported the synthesis of thermos-responsive of poly(N-isopropyl-acrylamide-cohydroxymethyl acrylamide)-shell–MSNs for controlled drug release[10]. Chen et al. reported the preparation of intelligent drug delivery system based on MSNs-coated with an ultra-pH-sensitive polymer and poly(ethylene glycol)[29]. Chang et al. have prepared thermo and pH dual responsive, poly(N-isopropylacrylamide-co-methacrylic acid) shell-coated, magnetic-MSNs for controlled drug release[30].

Little work has been focused on the synthesis secondary amine-functionalized polymer grafted on surfaces [31,32]. Morse and coworkers reported the preparation of latex particles from 2-(tert-butylamino)ethyl methacrylate (TBAEMA) using aqueous emulsion polymerization[33]. It has been reported the preparation of PTBAEMA-functionalized polyolefin fibers via ATRP and an azide-functional initiator [31,34]. Ding et al. reported the synthesis of PTBAEMA brushes from a planar surface via living radical polymerization[35]. It has been reported the growth of uniform PTBAEMA brushes from planar surfaces using SI-ATRP and studied the pH-responsive behaviour of these linear brushes[32].

Alswieleh et al. reacted a polymeric diisocyanate with secondary amines in PTBAEMA chains when immersed in a good or bad solvent, to either uniform crosslink or surface cross-link[32]. The behaviour of the resulting brushes was observed to be strongly dependent on the spatial location of the cross-linking reaction. Cheng et al. reported the growth of poly(2-dimethylamino)ethyl methacrylate) (PDMA) brushes from planar substrates[36]. Surface quaternization of the PDMA was achieved by conducting the polymer to 1-iodooctadecane in a poor solvent (n-hexane), producing pH-responsive brushes with hydrophobic upper surface. Madsen et al. prepared poly(cysteine methacrylate) (PCysMA) on glass and used THF (poor solvent) to cause collapse of the PCysMA brushes to achieve selective derivatisation of amine groups with glutaraldehyde at the interface between the collapsed brush and solvent, facilitating attachment of aminobutyl(nitrile triacetic acid) (NTA)[37].

In this study, mesoporous silica nanoparticles (MSNs) were prepared with relatively high surface area (~1000 m^2^ /g), and pore size of ~6.0 nm. Uniform PTBAEMA brushes were grown from MSNs surfaces using surface ATRP. The pH-responsive behaviour of these brushes was characterized using dynamic light scattering and compared to reacted polymers with iodoethanol in alkali media. Spatial confinement can be achieved as the reaction time passes. It is expected at the beginning, the reaction occurs to the upper surface of the collapsed brush. As the reaction time passes, iodoethanol reacts uniformly throughout the swollen brush layer. In principle, spatial control should affect the pH-responsive behaviour of these brush layers. This hypothesis is examined using various characterization techniques, including dynamic light scattering (DLS), thermal gravimetric analysis (TGA) and X-ray photoelectron spectroscopy (XPS).

## Experimental

### Materials

Deionized water was obtained using an Elga Pure Nanopore 18.2 MΩ system. 3-Aminopropyltriethoxysilane (APTES, >98%), 2-bromoisobutyryl bromide (BIBB, 98%), 2-iodoethanol (99%), triethylamine (TEA, 99%), 2-(tert-butylamino)ethyl methacrylate (TBAEMA, 97%), N-cetyltrimethylammonium bromide (CTAB, 98%), tetraethylorthosilicate (TEOS, 98%), copper(I) chloride (>98%), copper(II) bromide (>99%), 2,2ʹ bipyridine (>99%), methanol (99.8% HPLC grade), ethanol (99.8%, HPLC grade), isopropyl alcohol (analytical grade), toluene (analytical grade), dichloromethane (DCM, HPLC grade), and ammonium hydroxide (28 wt%), were purchased from Sigma-Aldrich. Hydrochloric acid (HCl) and were obtained from Fisher Scientific. All the chemicals were used as received. Copper(I) choride was stored under vacuum prior to use. TBAEMA was treated with basic alumina to remove inhibitor and stored at 5°C before use.

### Mesoporous silica preparations and functionalization

#### Synthesis of mesoporous silica nanoparticles

Typically, 1.0 g of CTAB was dissolved in 160 mL of deionized water under stirring. Then, 7.0 mL of concentrated ammonia water (28 wt %) was added, forming a clear solution. After that, a mixture solution of n-hexane (20 mL) and TEOS (5 mL) were added into the solution dropwise within 30 min under continuous stirring. As the reaction proceeds at 35°C, a homogeneous milky colloidal solution was gradually formed under continuous stirring (200 rpm). After stirring for 12 h, the product was collected by centrifugation and washed with deionized water and ethanol. After that, the collected solid sample was dried in an oven at 100°C for 2 h.

#### Synthesis of 3-aminopropyl-functionalized MSNs (AP-MSNs)

Amino modification of the silica surface was performed by suspending the obtained nanoparticles (1.5 g) in a solution of APTES (2.5 mmol) in dry toluene (50.0 mL) and the resulting mixture was heated under reflux (130°C) for 24 h. The nanoparticles were collected by centrifugation, washed twice with toluene and five times with ethanol, and dried under vacuum.

#### Formation of AP-MSNs nanochannels

AP-MSNs samples were treated with solvent extraction to remove CTAB templates by re-dispersing 1.5 g of the sample in methanol (160 mL), to which a concentrated aqueous solution of HCl (12 M, 9 mL) was added, and the mixture was heated under reflux for 24 h. The sample was collected by centrifugation, followed with washing with ethanol 6 times, and finally vacuum dried overnight.

#### ATRP initiator on MSNs outer surface (BiBB-MSNs)

AP-MSNs (1.0 g), DCM (25.0 mL), and triethylamine (1.5 mL, 11 mmol) were added into a 100 mL flask, and then 2-bromo-2-methylpropionyl bromide (1.2 mL, 10 mmol) in 5 mL of DCM was added dropwise into the mixture at room temperature. The resulting mixture was stirred at room temperature for 24 h. The solid was then separated by filtration and washed three times with DCM. The obtained product was redispersed in 20 mL of DCM by ultrasonic processing, filtered, and washed three times with DCM and five times with ethanol, and dried under vacuum.

#### ATRP synthesis of PTBAEMA brushes on MSNs

BiBB-MSNs (200 mg) was mixed TBAEMA (2.7 g, 15 mmol), isopropanol (IPA; 4.0 mL) and water (1.0 mL) at 40°C, and deoxygenated for 30 min. Bipy (30.0 mg, 0.2 mmol) and Cu(II)Br_2_ (2.2 mg, 0.01 mmol) were added to the solution and deoxygenated for 15 min, after which Cu(I)Cl (2.0 mg, 0.02 mmol) was added to the reaction mixture. The surface polymerization of TBAEMA was allowed to proceed at 40°C under nitrogen atmosphere for 2 h. Then, the product was washed with IPA, ethanol, and dried under vacuum.

#### Surface modification of PTBAEMA brushes

The surface of PTBAEMA brush layer was modified using iodoethanol. PTBAEMA-MSNs (0.5 g) were suspended in water at pH 8.5 and 40°C. Iodoethanol (0.5 g) was added to the solution under stirring. The solid was separated by centrifugation and washed with water, ethanol, and dried under vacuum.

### Measurement and characterization

#### Surface area analysis

The surface area of silica nanoparticulate materials used in this study was measured using nitrogen physisorption isotherms on a Micromeritics Gemini 2375 volumetric analyzer. Each sample was degassed prior to analysis for 6 h at 150°C. The Brunauer–Emmett–Teller (BET) surface areas were calculated using experimental points at a relative pressure (P/P^o^) of 0.05–0.25. The total pore volume was calculated from the N_2_ amount adsorbed at the P/P^o^ of 0.99 for each sample and the average pore size distribution of the materials was calculated using the Barrett–Joyner–Halenda (BJH) model. *FTIR Spectroscopy*: Infrared spectra of all samples were obtained in KBr pellets in the 4000–400 cm^−1^ region with a resolution of 4 cm^−1^, using a Thermo Scientific Nicolet iS10. *Elemental analysis (EA*): Elemental analysis was carried out using a Perkin Elmer Series II-2400 analyzer. *Scanning Electron Microscopy (SEM)*: SEM images were collected using JEOL JSM-6380 LA scanning electron microscope. The dried samples were directly used for the observation without any treatment. *Transmission Electron Microscopy (TEM)*: A drop of dilute sample suspension in ethanol was placed on a copper grid with a thin polymer coating and dried at room temperature prior to the measurement. A JEOL JEM-1230 transmission electron microscope was used for TEM imaging. *Thermogravimetric analysis (TGA)*: TGA analyses were carried out on an SII TGA 6300 instrument with a heating rate of 10°C/min under N_2_. *Dynamic Light Scattering (DLS)*: The intensity-average hydrodynamic diameter of fabricated nanoparticles was determined using a Malvern Zetasizer instrument. Aqueous dispersions were analyzed using disposable plastic cuvettes.

#### Cytotoxicity

Alamar blue ((AbD Serotec, Raleigh, NC, USA)) was used to determine the cytotoxicity of the nano-silica particles for both coated and non-coated particles according to manufacturer’s instructions. Human mesenchymal stem cells (TERT-20 hMSCs) were used to test for the cytotoxicity. Cells were cultured in 96 well plates in Dulbecco’s Modified Eagle’s Medium (DMEM) with 4500mg/L glucose, 1mM sodium pyruvate and 4mM glutamine (Cat. No. TDMEM-111,014, UFC Biotech, Riyadh, KSA) supplemented with 10% fetal bovine serum (FBS), 1% penicillin-streptomycin and 1% non-essential amino acids (all from Gibco, Invitrogen, USA). Cells were exposed to the nanoparticles for 4, 24 and 48 h at three different concentrations of 10,50 and 100 µg/ml. After designated exposure times, 20 µl of alamar blue reagent was added to the tested wells and incubated at 37°C for 1 h in the dark by covering the plate with foil. After that, the plates were read in a plate reader (BioTek Inc., Winooski, VT, USA) at an excitation wavelength of 530nm and an emission wavelength of 590nm. Control sample was hMSCs alone without the nanoparticles and the viability of the control was considered 100% viability and compared to the exposed cells. Cell viability was measured using the following formula: [Cell Viability, % = 100 x (corrected FI 590 of the cells treated with nanoparticles/corrected FI 590 of untreated control cells)], where FI 590 = Fluorescent Intensity at 590 nm emission (560 nm excitation). Software used for data analysis were Microsoft excel and GraphPad Prism 6.0.

**Scheme 1.**
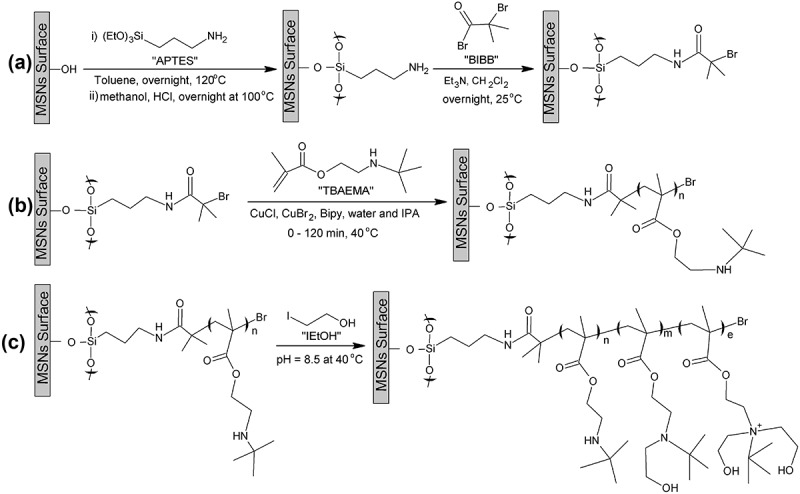
(A) Schematic representation of the formation of an amide-based ATRP Initiator Layer on mesoporous silica nanoparticles (MSNs) surface. (B) Synthesis of PTBAEMA brush from an initiator-functionalized MSNs surface via ATRP in water/isopropyl alcohol at 40 °C. (C) Suggested surface modification of PTBAEMA brush layer when using iodoethanol at pH 8.5 and 40 °C.

### Results and discussion

The MSN-PTBAEMA was fabricated according to Scheme 1. MSNs were synthesized based on a similar Stöber process. MSNs were functionalized with APTES before CTAB removal in order to avoid functionalization in the internal surface of the pores. The template molecules were then removed by washing the nanoparticles with acidic solution in methanol. The MSN-Br was obtained by reaction of amino groups in MSN-NH_2_ with 2-bromo-2-methylpropionyl bromide, and then it was used to grow PTBAEMA on external surface of MSNs via SI-ATRP.

**Figure 1.**
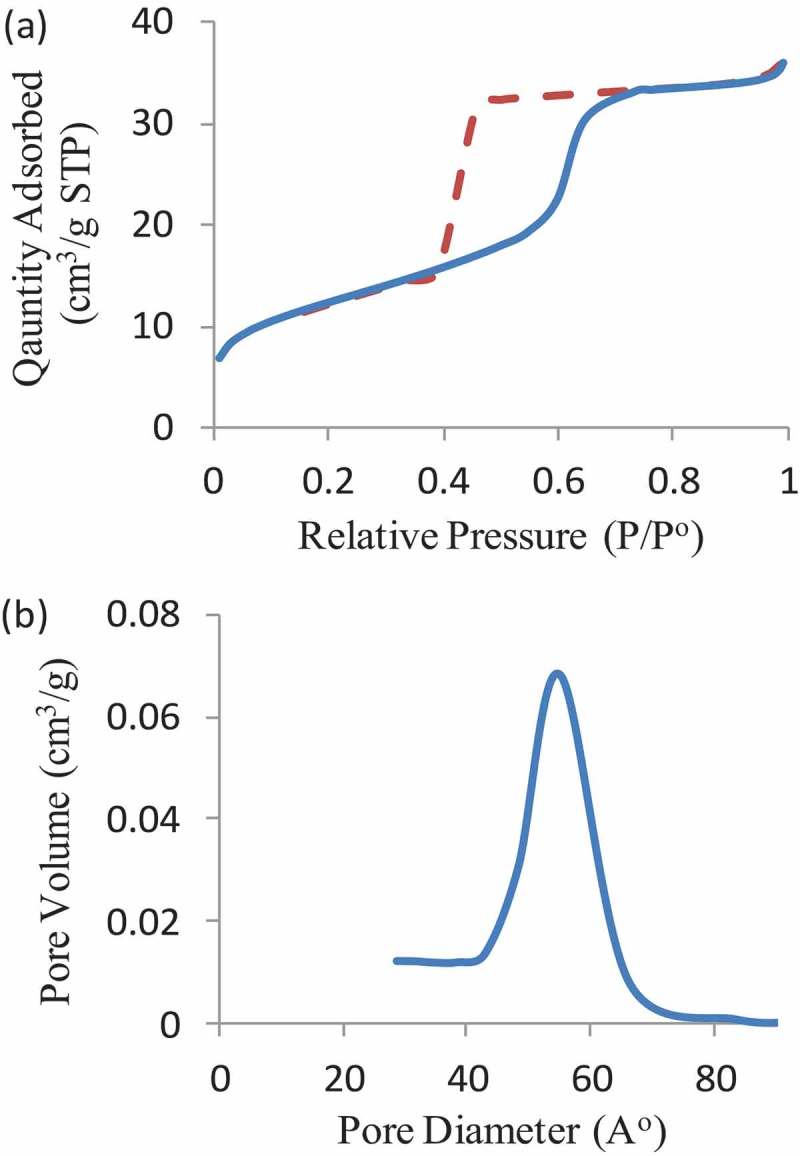
(a) Nitrogen adsorption isotherms of the MSN-Br sample. (b) BET pore size distribution patterns of the MSN-Br initiated.

Fabricated nanoparticles were characterized using nitrogen adsorption measurement to specify the mesopore characters of MSNs and MSN-Br, that is, the average pore diameter, surface area, and pore volume. The N_2_ adsorption isotherms for nanoparticles were found to be Type IV, confirming their mesoporous nature (Figure 1(a)). The BET surface area and pore volume of MSNs are 998 m^2^ g^−1^ and 1.5 cm^3^ g^−1^, respectively. In comparison, the BET surface area of initiated nanoparticles is 670 m^2^ g^−1^, and the pore volume is 0.94 cm^3^ g^−1^. The pore size of initiated samples was about 6 nm with a relatively narrow pore size distribution (Figure 1(b)).

**Figure 2.**
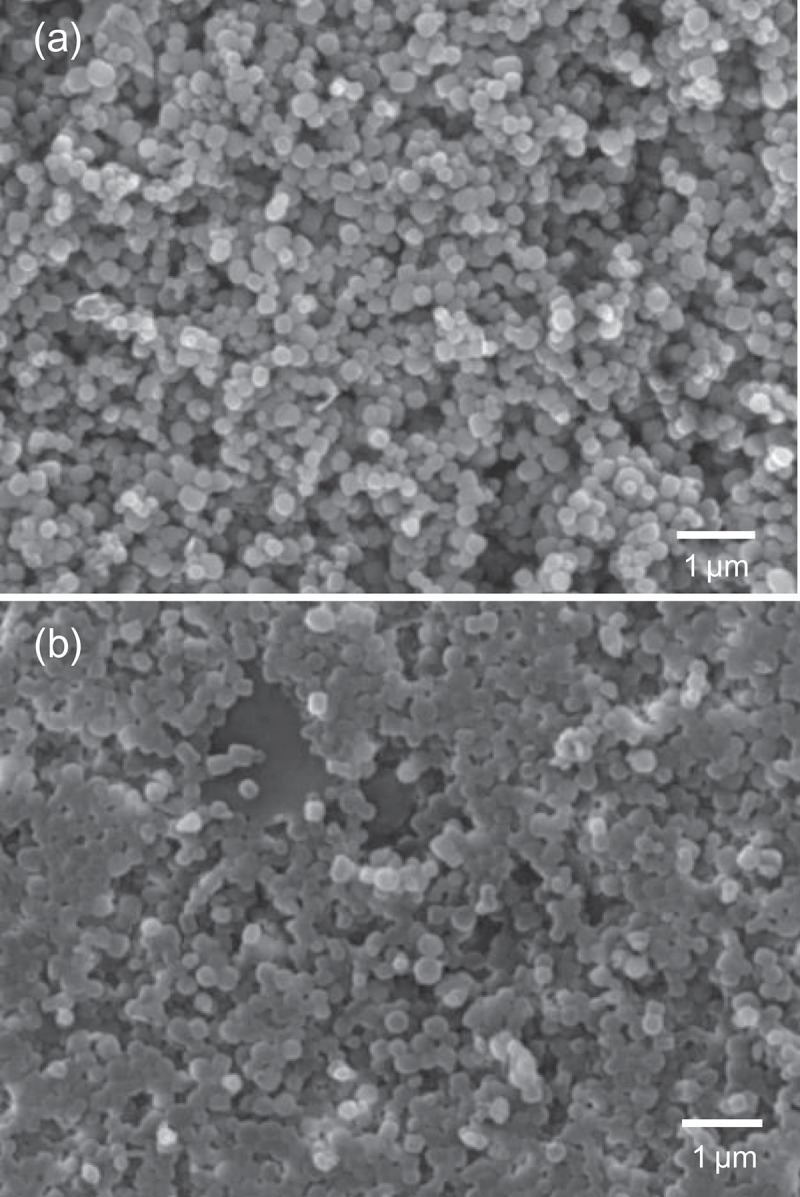
SEM images of mesoporous silica nanoparticles. (a) SEM image before polymerization. (b) SEM image after polymerization.

The morphology (size, shape and distribution) of the colloidal mesoporous silica nanoparticles was observed by scanning electron microscopy (SEM) and transmission electron microscopy (TEM). SEM images of nanoparticles before and after the surface-initiated polymerization of PTBAEMA were shown in Figure 2. SEM image indicates that the MSNs-Br sample consists of nanosphere structures. As shown in Figure 2(a), the polydispersity of MSN-Br nanoparticle size range of 150–250 nm. As can be seen in Figure 2(b), each silica nanoparticle is coated with outer layer of PTBAEMA brushes, compared with MSNs-Br, consistent with the observations of Zhiping Du[38].

**Figure 3.**
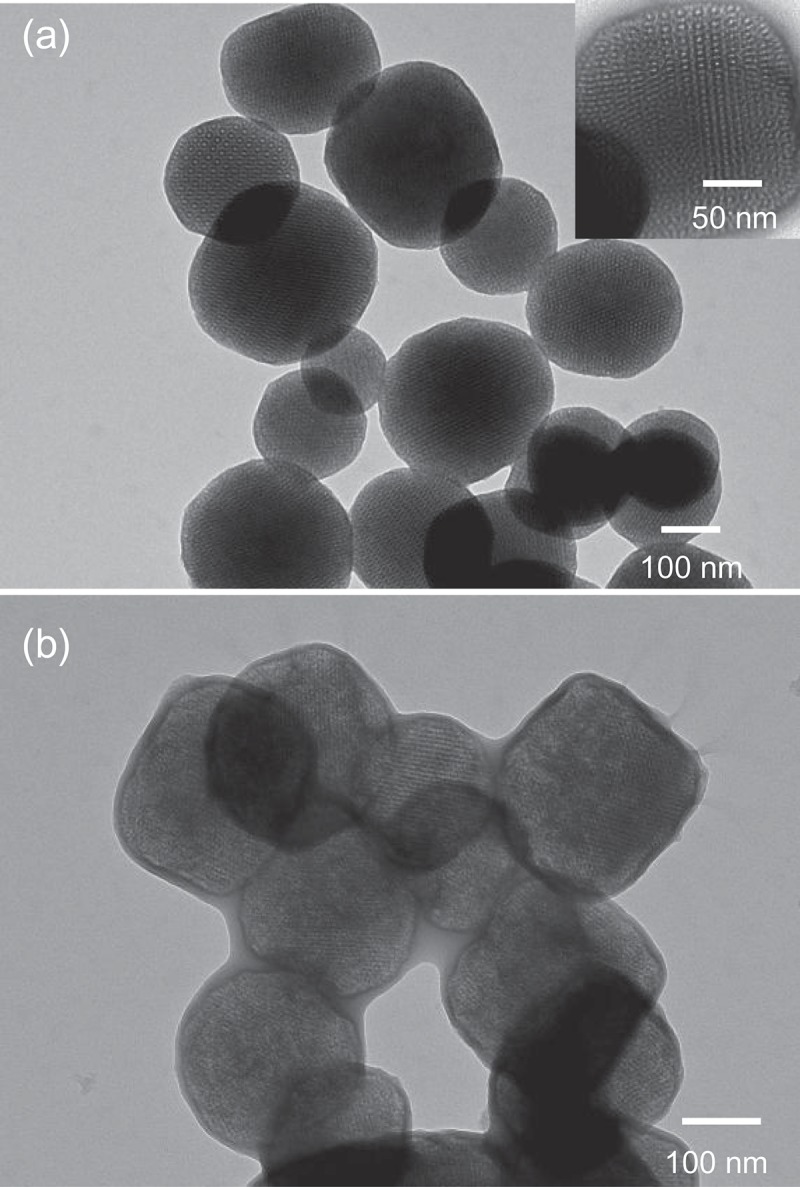
TEM images of mesoporous silica nanoparticles. (a) TEM image before polymerization. (b) TEM image after polymerization.

TEM images of bare MSN-Br and MSN–PTBAEMA composites are showed in Figure 3. As can be seen from Figure 3(a), the MSN-Br nanoparticles are highly dispersed and have regular morphology with a diameter ranging between 150 nm and 250 nm, which is in agreement with the SEM results and nothing can be seen around the nanoparticles. The mesopores size was ca. 6 nm, as estimated from the TEM image which agrees with BET surface analysis. The pore size is larger than those of typical mesoporous silica nanoparticles, due to the effect of n-hexane. After grafted with PTBAEMA, the observed diameter of MSN-PTBAEMA became larger and clear core/shell structures were formed (Figure 3(b)). This implied that the polymer chains had been attached to the surface of MSNs.

Infrared spectroscopy was used to verify that molecules had been successfully attached to the surface of MSNs. The FTIR spectra of MSNs, MSN-NH_2_, MSN-Br, MSN–PTBAEMA and surface-quaternized of MSN–PTBAEMA. FTIR spectra show wide bands at 1240–1030 cm^−1^ which attributed to the stretching of Si–O–Si bands of the condensed silica network. Peak at ~810 cm^−1^ was assigned to stretching vibration of Si–O. Peaks at ~1490 cm^−1^ and ~2930 cm^−1^ were assigned to C–H as – CH_2_ – in CTAB. After template extraction, the peaks at ~1490 cm^−1^ and 2930 cm^−1^ were almost disappeared, indicating the removal of CTAB template. Peaks at ~695 cm^−1^, ~1490 cm^−1^, ~1610 cm^−1^ and ~1565 cm^−1^ were assigned to APTES modification. After amidation, a doublet peaks were found in the vicinity of ~1380 cm^−1^, characteristic absorptions due to the isopropyl methyl groups deformation in 2-bromoisobutyrate. For MSN-PTBAEMA, the peak at ~2980 cm^−1^ was ascribed to the C–H antisymmetric stretching vibration, and the peak at ~2850 cm^−1^ is the – CH_2_ – stretching vibration. The absorption band at ~1730 cm^−1^ belongs to – C = O stretching. The presence of these peaks confirmed that the MSN-PTAEBMA has been prepared successfully. FTIR spectra were noticed to be similar to the MSN-PTAEBMA after the reaction with iodoethanol, indicating the successful reactions with amine groups.

Elemental analysis was used to estimate the amount of molecules (Lo) attached to the surfaces of the MSN-Br, MSN-PTEABMA and Surface-quaternized of MSN-PTBAEMA samples using the measured percentage of nitrogen; ass shown in the following equation:

$$L_{o}=\frac{\%N}{nitrogen\;atomic\;weight}\times \;10$$

The L_o_ values were calculated and demonstrated in Table 1.

**Table 1.** Elemental analysis data obtained for the modified mesoporous silica nanoparticles.

Sample ID	N %	C %	H %	L_o_(mmol/g)^a^
Surface initiated (MSN-Br)	1.74	7.16	1.93	1.24
PTBAEMA brushes	3.87	21.64	3.94	2.76
Surface-quaternized PTBAEMA brushes (for 1h)	3.79	22.81	3.48	2.70
Surface-quaternized PTBAEMA brushes (for 3h)	3.61	24.14	4.69	2.57
Surface-quaternized PTBAEMA brushes (for 24h)	3.73	24.38	4.27	2.66

^a^ Functionalization degree (in millimoles of ligand per gram of functionalized silica).

**Figure 4.**
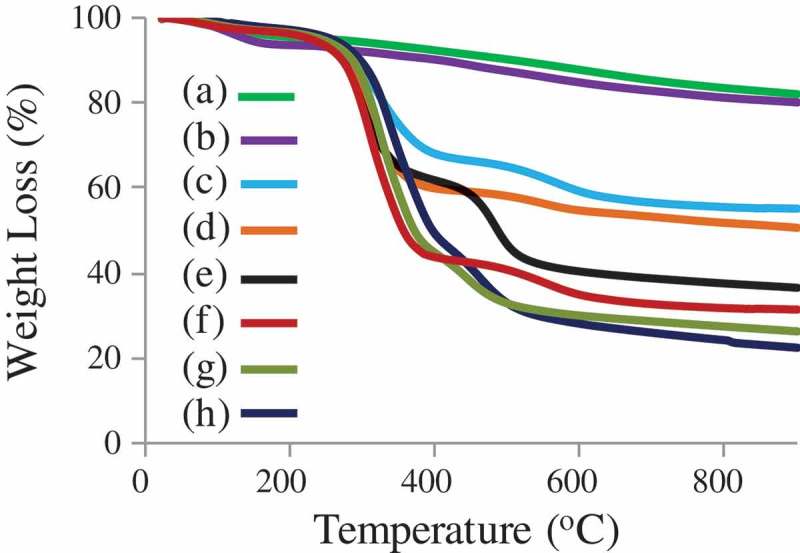
Thermogravimetric analysis (TGA) of (a) MSN-NH_2_ (after CTAB extraction), (b) MSN-Br (c) MSN + CTAB, (d) MSN-NH_2_ + CTAB, (e) MSN-PTAEBMA, (f) Surface-quaternized of MSN-PTBAEMA (for 1 h), (g) Surface-quaternized of MSN-PTBAEMA (for 3 h), and (h) Surface-quaternized of MSN-PTBAEMA (for 24 h).

The successful surface modification of the mesoporous silica was evaluated by thermogravimetric analysis (TGA), when heating under a nitrogen flow at a heating rate of 10°C min^−1^ to 900°C (Figure 4). TGA indicated that the weight loss at ~550°C was ~40% for bare MSN before CTAB extraction and ~44% after surface modification with APTES (MSN-NH_2_ with CTAB). The weight loss was ca. 10 wt% for both MSN-NH_2_ and MSN-Br. The result revealed that the content of – NH_2_ in the surface of MSNs were ca. 0.50 mmol/g. The mass loss of MSN-PTBAEMA was far less than of MSN-Br, at ~550°C was ~60%. TGA indicated that PTBAEMA was grafted onto MSNs surface with high density and the content of the monomer was estimated to be ca. 2.7 mmol/g. The weight loss was ca. 5% when the surface of PTBAEMA brushes was quaternized for 1 h using iodoethanol, and ca. 10% when the reaction is performed for 3 h and 24 h, compared to MSN-PTBAEMA. These results suggest that ca. 10% of amino group on the polymer chains was reacted iodoethanol when the reaction preformed for 1 h, and ca. 20% was reacted when the reaction performed for 3 h and 24 h.

**Figure 5.**
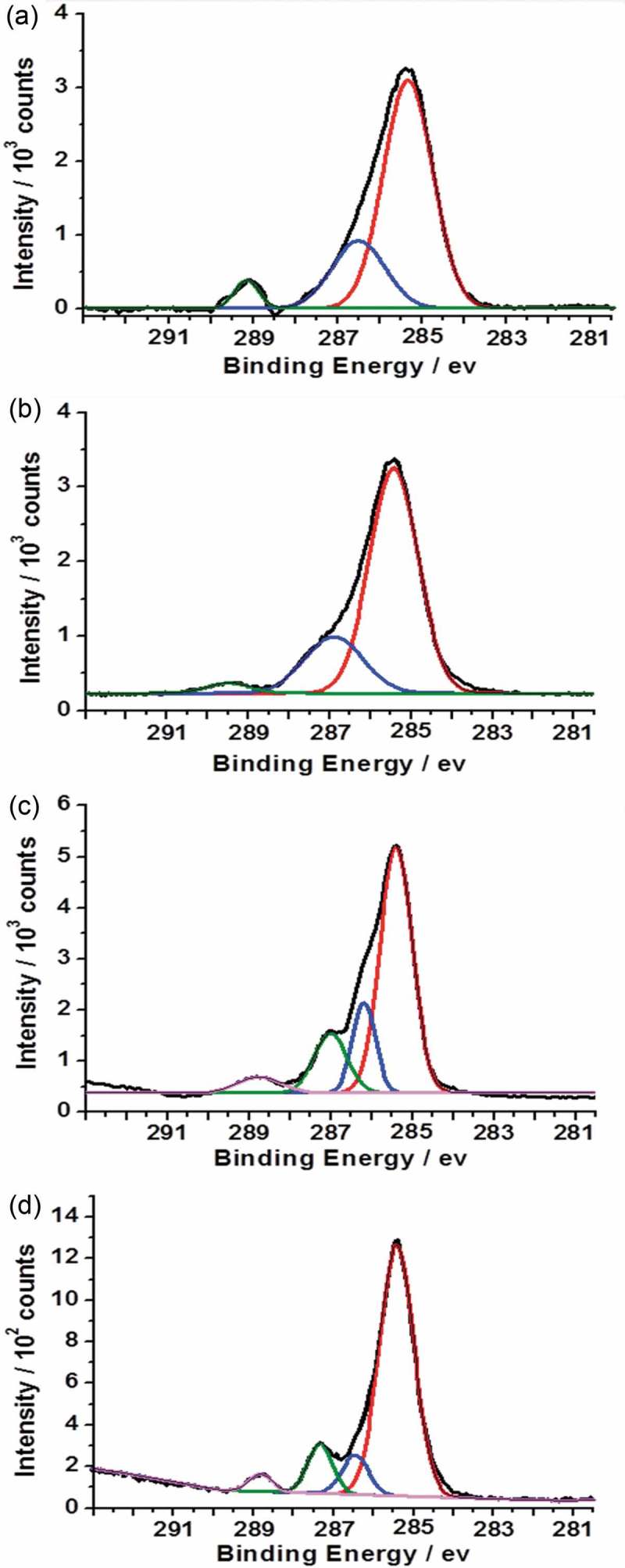
X-ray photoelectron core-line spectra recorded for a series of PTBAEMA brushes grafted on MSNs. (a) C1s spectrum obtained for MSN-PTAEBMA. (b) C1s spectrum obtained for surface-quaternized of MSN-PTBAEMA (for 1 h). (c) C1s spectrum obtained for surface-quaternized of MSN-PTBAEMA (for 3 h). (d) C1s spectrum obtained for surface-quaternized of MSN-PTBAEMA (for 24 h).

The C1s spectrum of PTBAEMA brush grafted on MSNs is shown in Figure 5(a). The peaks corresponding to hydrocarbon carbon atoms were fitted at 285.0 eV (−C−C−). The peaks corresponding to C−O bonds and to the carbon atoms adjacent to nitrogen (−C−NH−) were fitted using a single component at 286.5eV. The ester carbonyl component was fitted at 288.9 eV. The peak areas after fitting were calculated to be 7.1:3.8:1, which are in reasonable agreement with the theoretical ratio of 6:3:1. As expected, the C1s spectrum of a PTBAEMA brush after reaction with iodoethanol for 1 h was observed to be similar to as prepared PTBAEMA brush (Figure 5(b)), due to the quaternization reaction is confined to the (near) surface. Given that the sampling depth of XPS is ca. 10 nm, only very small fraction of the C−O could be located within the XPS sampling depth. Surface-quaternized of MSN-PTBAEMA for 3 h and 24 h, led to an increase in the contribution from the C−O component (Figure 5(c,d)). Separate components were included to account for the C−N signal at 285.8 eV and the C−O signal at 286.5 eV. A substantial increase in intensity is observed for the C−O component at 286.5 eV, due to the presence of additional C−O bonds contributed by iodoethanol. These data are consistent with the hypothesis that iodoethanol reacts uniform within the brush layer as the time passes.

In previous studies, acid titration indicates a pKa of around 7.8 for PTBAEMA brushes (weak polyelectrolyte) in aqueous solution[32]. PTEABMA chains become protonated on the addition of HCl; and they stretch away from the surface to minimize the strong interchain electrostatic repulsive forces. In contrast, fully quaternized PTBAEMA exhibits no pH-responsive character. However, partially quaternized PTBAEMA lead to an intermediate pH-responsive behaviour.

**Figure 6.**
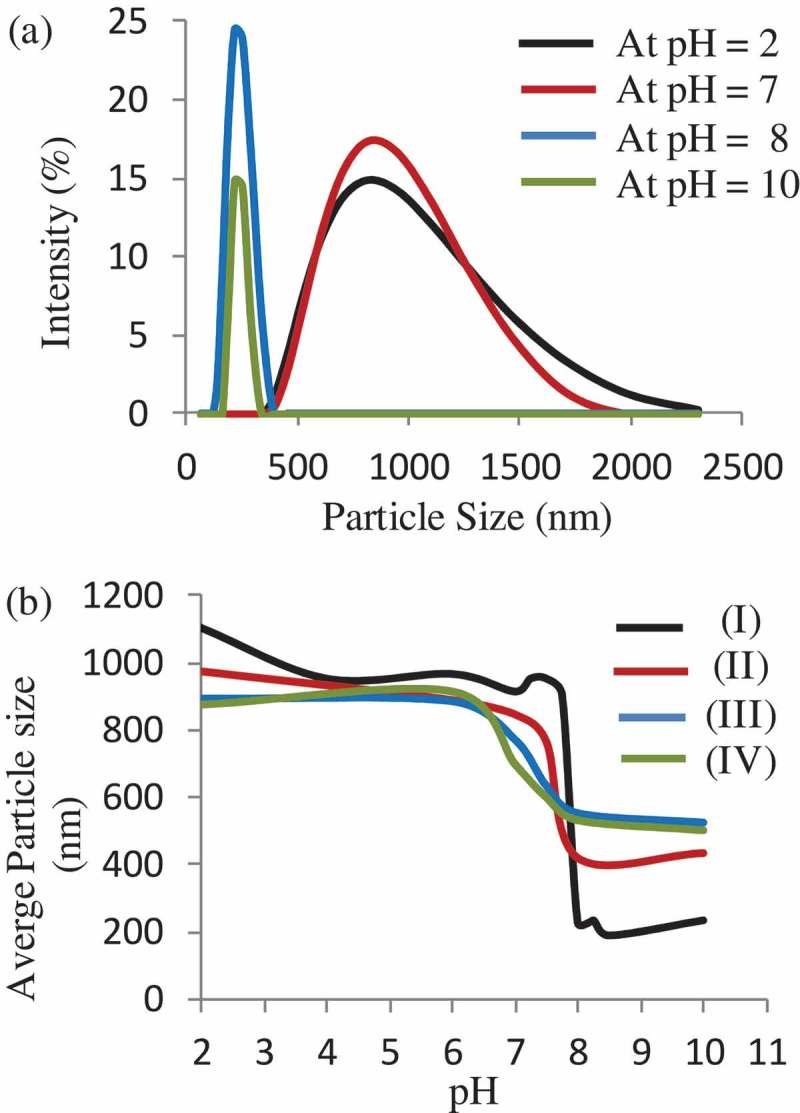
(a) Size distribution of MSN-PTBAEMA in buffer solution at different pH values. (b) The average particle size of MSN-PTAEBMA (I), Surface-quaternized of MSN-PTBAEMA (for 1 h) (II), Surface-quaternized of MSN-PTBAEMA (for 3 h) (III), and Surface-quaternized of MSN-PTBAEMA (for 24 h) (IV) at different pH values.

The pH-responsive behaviour of a PTEABMA precursor brushes and quaternized PTBAEMA brushes were investigated. The size distribution of the samples in aqueous buffers, and the average hydrodynamic diameter (Dh) at different pH values ranging from pH 2 to 11, were measured by dynamic light scattering (DLS), as shown in Figure 6. As can be seen, the Dh of the modified MSNs with PTEABMA precursor brushes ranged from 400 to 2000 nm at pH < 7 (Figure 6(a)). The PTEABMA precursor chains can induce chain−chain repulsions; thus, a hydration layer is arising on the surface of MSN nanoparticles and resulting in a larger particle diameters distribution. The hydrodynamic diameter of MSN@ PTBAEMA at pH >8 was found to be between 200 − 400 nm, due to PTEABMA chains are hydrophobic at this media.

As expected, the MSN-PTBAEMA becomes fully protonated at pH <8 and attains its maximum swollen thickness of approximately 1000 nm (see black data set in Figure 6(b)). The deprotonated PTBAEMA brush on MSNs adopts collapsed at ca. 250 nm. Surface-quaternized of MSN-PTBAEMA (for 3 h), and Surface-quaternized of MSN-PTBAEMA (for 24 h) exhibited pH-responsive behaviour in acidic media (pH < 7). When the particles immersed in alkaline buffer (pH > 8), the brushes exhibited a relatively collapsed conformation, approximately at 600 nm (green and blue data sets in Figure 6(b)). The quaternized of MSN-PTBAEMA exhibited that a somewhat lower pH (pH ∼ 6.5) was required to achieve the maximum height, compared to 7.8 for the PTEABMA precursor brushes. At first sight, the Surface-quaternized of MSN-PTBAEMA derivatized in alkali media for 1 h appears to exhibit intermediate behavior (see red data set in Figure 6(b)). It is clear that there is actually little difference between the PTBAEMA precursor brushes and the surface-quaternized PTBAEMA brushes for 1 h. The reason is that the quaternization is confined to the (near) surface, when the PTBAEMA precursor brushes was exposed to iodoethanol solution for 1 h.

**Figure 7.**
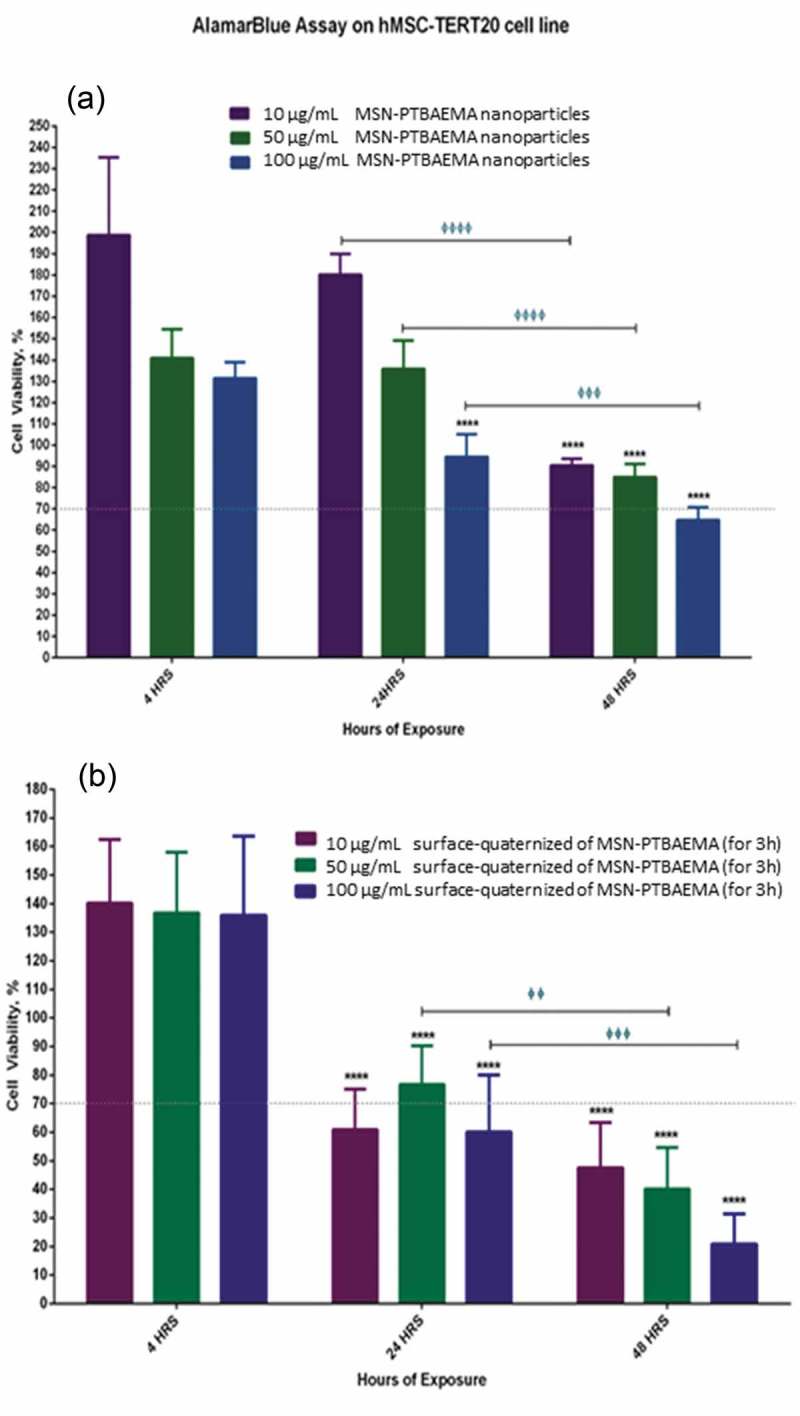
Illustrations of the effect of MSN-PTBAEMA nanoparticles on cell viability of hMSCs (a) and the effect of surface-quaternized of MSN-PTBAEMA (for 3 h) nanoparticles on cell viability of hMSCs (b), after being exposed to the nanoparticles for 4, 24 and 48 h, and Sideway bars indicate significance for the same concentrations at different exposure times and astride indicate the level of significance as follows: *P > 0.05, **P > 0.01, ***P > 0.001, ****P > 0.0001.

For analysis of results, 70% reduction in cell viability was considered to be toxic to the cells, as shown in Figure 7. MSN-PTBAEMA and surface-quaternized of MSN-PTBAEMA (for 3 h) samples were not toxic to cells after 4 h of exposure for all three concentrations of 10 µg/ml, 50 µg/ml and 100 µg/ml. When increasing the exposure of the MSN-PTBAEMA nanoparticles to 24 h, there was no significant reduction in viability for both the 10 and 50 µg/ml concentrations, however, the 100 µg/ml showed a slight reduction in cell viability but it did not reach the cytotoxic levels. Surface-quaternized of MSN-PTBAEMA (for 3 h) nanoparticles showed decreases in cell viability after 24 h of exposure with the 10 and 100 µg/ml concentration causing cell viability to drop to slightly toxic levels of nearly 60% cell viability and 50 µg/ml did not show cytotoxicity. After 48 h, cells exposed to MSN-PTBAEMA nanoparticles showed decrease in cell viability but did not reach the cytotoxic levels except for the 100 µg/ml that dropped to nearly 65% cell viability; however, the reduction for all three was significant compared to the 24-h exposure. Surface-quaternized of MSN-PTBAEMA (for 3 h) nanoparticles showed cytotoxicity after 48 h of exposure for three concentrations with the gradual decrease in cell viability for the 10, 50 and 100 µg/ml concentrations and also significant reduction when compared to the 24-hour exposure.

## Conclusions

PTBAEMA brushes have been grafted on MSNs surfaces via SI-ATRP at 20°C. DLS studies confirmed that highly swollen-protonated brushes are produced at low pH, with collapsed deprotonated brushes above approximately pH 7.7. PTBAEMA brushes can react with iodoethanol via their amine groups to be quaternized. XPS spectra confirmed the presence of the reacted iodoethanol within the PTBAEMA brush. Variation of the 2-iodoethanol reaction time enables the spatial location of surface quaternization. At 1 h reaction time, leads to surface quaternization of the collapsed brush layer. On the other hand, when the brushes are conducting to iodoethanol for 3 h and more, leads to relatively uniform reaction throughout the brush layer. This spatial confinement affects the pH-responsive behaviour of the brush, as judged by DLS studies. As prepared PTBAEMA brush swelling occurs at approximately pH 7.7, which is the same critical pH as that found in the previous studied. However, it is observed that this critical pH is slightly lower when the brushed was reposed to iodoethanol for 3 h and more.

## Supplementary Material

Supplemental Material

## References

[1] Beck JS, Vartuli J, Roth WJ, et al A new family of mesoporous molecular sieves prepared with liquid crystal templates. J Am Chem Soc. 1992;114(27):10834–10843.

[2] Wu S-H, Mou C-Y, Lin H-P. Synthesis of mesoporous silica nanoparticles. Chem Soc Rev. 2013;42(9):3862–3875. 23403864 10.1039/c3cs35405a

[3] Reich S-J, Svidrytski A, Höltzel A, et al Hindered diffusion in ordered mesoporous silicas: insights from pore-scale simulations in physical reconstructions of SBA-15 and KIT-6 silica. J Phys Chem C. 2018;122(23):12350–12361.

[4] Mal NK, Fujiwara M, Tanaka Y. Photocontrolled reversible release of guest molecules from coumarin-modified mesoporous silica. Nature. 2003;421(6921):350. 12540896 10.1038/nature01362

[5] Burkett SL, Sims SD, Mann S Synthesis of hybrid inorganic–organic mesoporous silica by co-condensation of siloxane and organosiloxane precursors. Chem Comm. 1996;11(52):1367–1368.

[6] Lu J, Liong M, Li Z, et al Biocompatibility, biodistribution, and drug‐delivery efficiency of mesoporous silica nanoparticles for cancer therapy in animals. Small. 2010;6(16):1794–1805. 20623530 10.1002/smll.201000538 PMC2952648

[7] Chen K-J, Chen H-L, Tang-C-C, et al Synthesis of silica/polypeptide hybrid nanomaterials and mesoporous silica by molecular replication of sheet-like polypeptide complexes through biomimetic mineralization. J Colloid Interface Sci. 2019;542:243–252. 30763891 10.1016/j.jcis.2019.02.014

[8] Tang F, Li L, Chen D Mesoporous silica nanoparticles: synthesis, biocompatibility and drug delivery. Adv Mater. 2012;24(12):1504–1534. 22378538 10.1002/adma.201104763

[9] Slowing II, Vivero-Escoto JL, Trewyn BG, et al Mesoporous silica nanoparticles: structural design and applications. J Mater Chem. 2010;20(37):7924–7937.

[10] Liu C, Guo J, Yang W, et al Magnetic mesoporous silica microspheres with thermo-sensitive polymer shell for controlled drug release. J Mater Chem. 2009;19(27):4764–4770.

[11] Wang Y, Yin M, Lin X, et al Tailored synthesis of polymer-brush-grafted mesoporous silicas with N-halamine and quaternary ammonium groups for antimicrobial applications. J Colloid Interface Sci. 2019;533:604–611. 30193147 10.1016/j.jcis.2018.08.080

[12] Liu R, Liao P, Liu J, et al Responsive polymer-coated mesoporous silica as a pH-sensitive nanocarrier for controlled release. Langmuir. 2011;27(6):3095–3099. 21314163 10.1021/la104973j

[13] Zhang X, Yang P, Dai Y, et al Multifunctional Up‐converting nanocomposites with smart polymer brushes gated mesopores for cell imaging and thermo/pH dual‐responsive drug controlled release. Adv Funct Mater. 2013;23(33):4067–4078.

[14] Barbey R, Lavanant L, Paripovic D, et al Polymer brushes via surface-initiated controlled radical polymerization: synthesis, characterization, properties, and applications. Chem Rev. 2009;109(11):5437–5527. 19845393 10.1021/cr900045a

[15] Husseman M, Malmström EE, McNamara M, et al Controlled synthesis of polymer brushes by “living” free radical polymerization techniques. Macromolecules. 1999;32(5):1424–1431.

[16] Matyjaszewski K, Miller PJ, Shukla N, et al Polymers at interfaces: using atom transfer radical polymerization in the controlled growth of homopolymers and block copolymers from silicon surfaces in the absence of untethered sacrificial initiator. Macromolecules. 1999;32(26):8716–8724.

[17] Perruchot C, Khan M, Kamitsi A, et al Synthesis of well-defined, polymer-grafted silica particles by aqueous ATRP. Langmuir. 2001;17(15):4479–4481.

[18] Zhao Y, Perrier S Synthesis of well-defined homopolymer and diblock copolymer grafted onto silica particles by Z-supported RAFT polymerization. Macromolecules. 2006;39(25):8603–8608.

[19] Hawker CJ, Bosman AW, Harth E New polymer synthesis by nitroxide mediated living radical polymerizations. Chem Rev. 2001;101(12):3661–3688. 11740918 10.1021/cr990119u

[20] Matyjaszewski K, Xia J Atom transfer radical polymerization. Chem Rev. 2001;101(9):2921–2990. 11749397 10.1021/cr940534g

[21] Schmaljohann D Thermo- and pH-responsive polymers in drug delivery. Adv Drug Deliv Rev. 2006;58(15):1655–1670. 17125884 10.1016/j.addr.2006.09.020

[22] Ji Y, Lin X, Zhang H, et al Thermoresponsive polymer brush modulation on the direction of motion of phoretically driven janus micromotors. Angew Chem. 2019;58(13):4184–4188. 30701642 10.1002/anie.201812860

[23] Minko S Responsive polymer brushes. J Macromol Sci. 2006;46(4):397–420.

[24] Draper J, Luzinov I, Minko S, et al Mixed polymer brushes by sequential polymer addition: anchoring layer effect. Langmuir. 2004;20(10):4064–4075. 15969399 10.1021/la0361316

[25] Grest GS, Murat M Structure of grafted polymeric brushes in solvents of varying quality: a molecular dynamics study. Macromolecules. 1993;26(12):3108–3117.

[26] Dong R, Lindau M, Ober CK Dissociation behavior of weak polyelectrolyte brushes on a planar surface. Langmuir. 2009;25(8):4774–4779. 19243153 10.1021/la8039384

[27] Treat ND, Ayres N, Boyes SG, et al A facile route to poly (acrylic acid) brushes using atom transfer radical polymerization. Macromolecules. 2006;39(1):26–29.

[28] Leggett GJ Tools for low-dimensional chemistry. Langmuir. 2019;35(24):7589–7602. 10.1021/acs.langmuir.8b02672 30365897

[29] Chen T, Wu W, Xiao H, et al Intelligent drug delivery system based on mesoporous silica nanoparticles coated with an ultra-pH-sensitive gatekeeper and Poly (ethylene glycol). ACS Macro Lett. 2015;5(1):55–58. 10.1021/acsmacrolett.5b00765 35668579

[30] Chang B, Sha X, Guo J, et al Thermo and pH dual responsive, polymer shell coated, magnetic mesoporous silica nanoparticles for controlled drug release. J Mater Chem. 2011;21(25):9239–9247.

[31] Thomassin J-M, Lenoir S, Riga J, et al Grafting of poly [2-(tert-butylamino) ethyl methacrylate] onto polypropylene by reactive blending and antibacterial activity of the copolymer. Biomacromolecules. 2007;8(4):1171–1177. 17348705 10.1021/bm0611228

[32] Alswieleh AM, Cheng N, Leggett GJ, et al Spatial control over cross-linking dictates the pH-responsive behavior of poly (2-(tert-butylamino) ethyl methacrylate) brushes. Langmuir. 2014;30(5):1391–1400. 24417283 10.1021/la403666y PMC4190050

[33] Morse AJ, Dupin D, Thompson KL, et al Novel Pickering emulsifiers based on pH-responsive poly (tert-butylaminoethyl methacrylate) latexes. Langmuir. 2012;28(32):11733–11744. 22794126 10.1021/la301936k

[34] Lenoir S, Pagnoulle C, Galleni M, et al Polyolefin matrixes with permanent antibacterial activity: preparation, antibacterial activity, and action mode of the active species. Biomacromolecules. 2006;7(8):2291–2296. 16903673 10.1021/bm050850c

[35] Ding S, Floyd JA, Walters KB Comparison of surface confined ATRP and SET‐LRP syntheses for a series of amino (meth) acrylate polymer brushes on silicon substrates. J Polym Sci A Polym Chem. 2009;47(23):6552–6560.

[36] Cheng N, Bao P, Evans S, et al Facile formation of highly mobile supported lipid bilayers on surface-quaternized pH-responsive polymer brushes. Macromolecules. 2015;48(9):3095–3103.

[37] Madsen J, Ducker R, Al Jaf O, et al Fabrication of microstructured binary polymer brush “corrals” with integral pH sensing for studies of proton transport in model membrane systems. Chem Sci. 2018;9(8):2238–2251. 29719697 10.1039/c7sc04424k PMC5897877

[38] Du Z, Sun X, Tai X, et al Synthesis of hybrid silica nanoparticles grafted with thermoresponsive poly (ethylene glycol) methyl ether methacrylate via AGET-ATRP. RSC Adv. 2015;5(22):17194–17201.

