# Supplementary material for: Surface modification of pH-responsive poly(2-(tert-butylamino)ethyl methacrylate) brushes grafted on mesoporous silica nanoparticles
Source: Des Monomers Polym. 2019 Dec 11;22(1):226–35. doi: 10.1080/15685551.2019.1699727 (PMC6913628; doi:10.1080/15685551.2019.1699727)
Supplement: Supplemental Material [file TDMP_A_1699727_SM9173.docx]

**Supplementary Materials:**

The following are available online at [www.mdpi.com/xxx/s1](http://www.mdpi.com/xxx/s1),

Figure S1: The FTIR spectra of the fabricated nanoparticles.
